# Interfacial Reactions in the Li/Si diffusion couples: Origin of Anisotropic Lithiation of Crystalline Si in Li–Si batteries

**DOI:** 10.1038/s41598-017-14374-0

**Published:** 2017-10-25

**Authors:** Yong-Seok Choi, Jun-Hyoung Park, Jae-Pyoung Ahn, Jae-Chul Lee

**Affiliations:** 1 Department of Materials Science and Engineering, Korea University, Seoul, 02841 South Korea; 2 Advanced Analysis Center, Korea Institute of Science and Technology, Seoul, 02792 South Korea

## Abstract

As opposed to the common understanding that diffusion into a cubic-structured single crystal is independent of its crystalline orientation, the diffusion of Li to crystalline Si (c-Si) is anisotropic, which acts as the major cause for the fracture of Si anodes in Li-ion batteries. Here, by conducting comprehensive/multi-scale simulation studies based on molecular dynamics and density functional theory, we elucidate how and why Li diffusion in c-Si is anisotropic. We found that Li ions diffuse to c-Si by following a particular atomic-scale space corresponding to the lowest value of the valence orbital in c-Si, causing Li ions to take a tortuous diffusion pathway. The degree of the tortuosity of the pathway differs depending on the crystallographic orientation of Si, and it acts as the major cause for anisotropic lithiation. We also develop a structural parameter that can quantitatively evaluate the orientation dependency of the lithiation of c-Si.

## Introduction

Spontaneous insertion of Li ions into crystalline Si anodes (also termed “lithiation”) causes the formation of various amorphous phases of lithiated Si (a-Li_*x*_Si) and is always accompanied by volume expansion of ~300%. However, the rate of the dimensional change of the crystalline Si (c-Si) differs depending on the crystallographic orientation^1–5^. This anisotropic lithiation causes the uneven swelling of c-Si and promotes the development of locally inhomogeneous residual stresses, which can induce the fracture of c-Si and reduce the cycle life of Li-ion batteries (LIBs)^3,6^. Extensive studies to prevent the fracture of c-Si during lithiation are underway^7–10^.

Anisotropic swelling of a material is known to arise from the anisotropy in the rate of the bulk diffusion, which is commonly observed in materials with ‘non-cubic’ structures^11^. From this perspective, the anisotropic lithiation in c-Si (diamond cubic) is rather unusual. Cui *et al*., based on the experimentally observed sharp interface^3,8^, reported that the reaction at the interface plays a key role in determining the overall lithiation process^12^. Subsequent numerical model studies on the interfacial region showed that the reaction rate at the interface differs depending on the crystallographic orientations^1,13,14^. This conceptual theory was later confirmed by experimental and simulation studies^15,16^. Recent TEM studies on the lithiation process of c-Si showed that the anisotropic lithiation arises from the selective peeling-off of {111} planes at a very thin layer of the interfacial region^15^. Although these earlier studies are instructive for demonstrating how different diffusivity causes anisotropic expansion, they did not explain why the Li diffusivity varies along the crystalline orientation of Si and thus the mechanism underlying the anisotropic interfacial reaction is largely unknown.

Recently, various computational efforts have been undertaken to provide the theoretical basis for the diffusion behavior at the interfacial region. Tachikawa *et al*. calculated the Li diffusion in two-dimensional (2D) carbonaceous anodes such as graphene^17^, bucky balls^18^, and amorphous carbon^19^ and demonstrated that Li ions tend to migrate along the pathway that can evade the highest occupied molecular orbital of the anode surface. Subsequent studies by Malyi *et al*.^20^, Jin *et al*.^21^, and Cubuk *et al*.^22^ calculated the energy barriers for diffusion to investigate the anode material with a high rate performance. Recently, Jung *et al*. calculated the reactions at the interface between Li_*x*_Si and Si and reported that the overall kinetics of lithiation are determined by the short-range atomic reactions occurring at the Li_*x*_Si/Si interface, which is therefore primarily responsible for the anisotropic behavior^23^. Despite the important findings on the diffusion behavior in various anode materials, they focus on the individual effects of the valence orbital, energy barriers, and short-range atomic reactions at the interface on the diffusion behavior. Furthermore, most previous analyses were based on first-principles calculations. Owing to the severe spatiotemporal limitation of this method, the models employed for previous calculations in many cases were too small to depict the general features of lithiation, and thus they are insufficient to explain the physical process regarding anisotropic lithiation behaviors occurring in three-dimensional (3D) Si anodes. In order to explain the physical process regarding anisotropic lithiation occurring in 3D Si anodes, an analysis of the combined effect of these parameters on the diffusion behavior in the Si anode is necessary.

In this study, using combined technique of classical molecular dynamics (MD) simulations and first-principles calculations, we performed a multi-scale study to elucidate how and why Li diffusion in 3D c-Si is anisotropic. This paper addresses three issues and is organized as follows: 1) We first resolve the diffusion process of Li ions in 3D c-Si using classical MD simulations performed on comparatively large-scale Li/Si diffusion couples with different orientations. 2) By combining the results of the MD simulations and first-principles calculations performed on the Li/Si diffusion couples, we next elucidate the presence of the particular diffusion pathway along which the diffusion of Li ions is favored and clarify why the Li diffusion in 3D c-Si is anisotropic. 3) We finally develop a structural parameter that can quantitatively evaluate the degree of anisotropy of the Li diffusion. By comparing the values of the developed parameter with the experimentally measured diffusivities along various orientations of c-Si, we reveal that the configuration of the valence orbital is a dominant factor determining the anisotropic lithiation.

## Methods

### Molecular Dynamics Simulations

Classical MD simulations employing the reactive force field (ReaxFF) potential^24,25^ were performed to resolve the initial stage of diffusion at the Li/Si interface. This technique can also provide the statistically meaningful description on the diffusion pathway of Li ions on comparatively large-scale c-Si at 300 K, which cannot feasibly be obtained via first-principles calculations alone. Although the ReaxFF potential used for the present MD simulations has already been extensively validated against a large set of experimental properties^3,6^ and *ab initio* data^25,26^, the reliability of the potential was tested again by comparing the dissociation energies of Si-Si bonds associated with lithiation with the predicted values^25,27^. In general, this potential well describes the experimental phenomena observed during the lithiation process^24,25,28,29^, as reported in previous works^3,6^.

**Figure 1:**
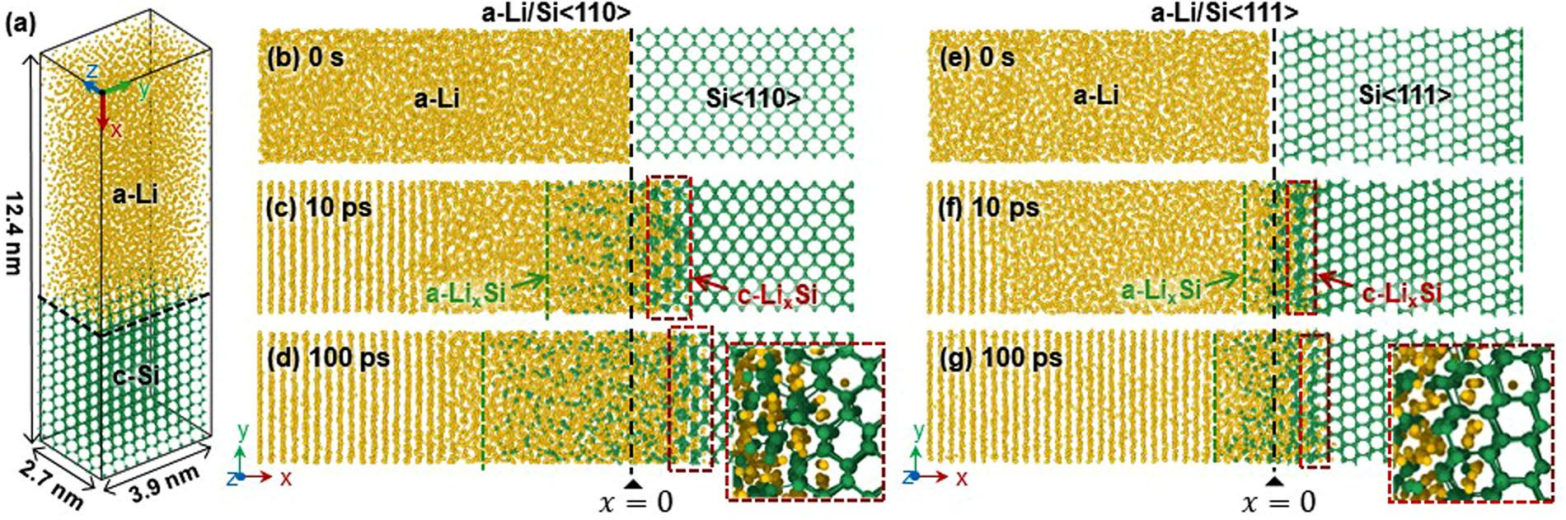
(**a**) Representative full-scale 3D calculation cell of the a-Li/c-Si diffusion couple used for the classical MD simulations. The lithiation process is simulated in the diffusion couples with the c-Si slab aligned along the crystallographic orientation of (**b**) <110> and (**e**) <111>. Snapshots captured from the (**b**–**d**) a-Li/Si<110> and (**e**–**g**) a-Li/Si<111> diffusion couples calculated at various times, showing the structural evolution at the a-Li/c-Si interface and the volume expansion of the c-Si slab. The insets of (**d**) and (**g**) correspond to the magnified views of the diffusion front, showing that lithiation proceeded by the formation of a thin layer of c-Li_*x*_Si.

To study the general features of the anisotropic lithiation of c-Si, we prepared computationally generated Li/Si diffusion couples by attaching an amorphous Li (a-Li) slab to two different c-Si slabs whose respective orientations were aligned along the <110> and <111> directions so that the c-Si (110) and (111) surfaces were in contact with the a-Li slab to construct the a-Li/Si<110> and a-Li/Si<111> diffusion couples, respectively (Fig. 1). Two types of boundary conditions were applied to the surfaces of the diffusion couples. Periodic boundary conditions were first imposed on the diffusion couples along the transverse directions (the *y*- and *z*-axes in Fig. 1) of the diffusion couples to account for the effect of the orientation of c-Si while eliminating the surface effects on the Li diffusion. The reflective boundary condition was applied to the free-surface side (the *x*-axis in Fig. 1) of the a-Li slab to keep the Li atoms from exiting the simulation box to prevent the unwanted diffusion behaviors occurring at the free boundary of the calculation cell while reducing the computation time. The two layers of Si atoms on the free surface of the c-Si slab were kept fixed, whereas the rest of the system was allowed to equilibrate. The simulations for Li diffusion into c-Si were performed in the micro-canonical ensemble with a time step of 1 fs using the ReaxFF potential^25^ implemented in the LAMMPS package^30^. The structure of the a-Li/c-Si diffusion couple was relaxed using a conjugate gradient method until the system attains an equilibrium state at 300 K. During this initial relaxation process, the inter-diffusion between Li and Si was prevented by fixing the constituent atoms near the interface. Within the NVT ensemble, the relaxed a-Li/c-Si diffusion couples were then equilibrated using a Nosé–Hoover thermostat to allow spontaneous lithiation at a constant temperature of 300 ± 6 K. All other variables and conditions used in the present simulations are summarized in Table 1.

**Table 1:** Types of simulation parameters and their values used in MD simulations.

Simulation parameters	Values
Numbers of Li atoms	6,000 atoms
Numbers of Si atoms	2,400 atoms
Size of the Li slab	7.8 × 3.9 × 2.7 nm (periodic boundaries on the y- and z- axes)
Size of the Si slab	4.6 × 3.9 × 2.7 nm (periodic boundaries on the y- and z- axes)
Energy minimization algorithm	conjugate gradient (CG) algorithm
Energy minimization cut-off	10^−8^ eV
Time step	1 fs
Total steps	100,000 steps

### First-principles calculations

In order to confirm the presence of the particular diffusion pathway along which the diffusion of Li ions is favored, the valence orbital of c-Si (diamond cubic) was calculated using first-principles calculations. The calculation cell was constructed to a 2 × 2 × 2 supercell structure prepared from the unit cell of a Si crystal. Periodic boundary conditions were enforced along the *x*-, *y*-, and *z*-directions. The calculations were performed with the plane-wave basis set and projector augmented wave (PBE-PAW) pseudopotentials^31,32^, as implemented in Quantum Espresso^33^. The *k*-point mesh was set to 8 × 8 × 8 using the Monkhorst–Pack scheme for the Brillouin zone sampling. The energy cutoff was 750 eV and convergence in the energy was achieved down to 10^−4^ eV.

To understand the mechanism of the Li diffusion and its orientation dependency, we calculated the energy barriers required for the Li diffusion using the nudged elastic band (NEB) method^34^ by considering the changes in the free energy of the Li ion during diffusion into c-Si with different crystallographic orientations. The NEB method adjusts the intentionally selected initially diffusion pathway of the Li ion so that the Li ion can travel along the pathway that costs the least energy. In order to calculate the energy barriers for the Li diffusion, it is therefore necessary to prepare the initial diffusion pathway of the Li ion and the c-Si through which the Li ion migrates. For this purpose, we first constructed two different c-Si slabs with their respective surface normal vectors parallel to <110> and <111> (hereinafter denoted as Si<110> and Si<111> slabs). The Si<110> and Si<111> slabs were modeled as the eight-layered (110) and (111) planes of c-Si, and a 30-Å-thick vacuum layer was added above the free surface. We then placed a Li ion in the c-Si slabs such that the initial diffusion pathway of the Li ion for NEB calculations was consistent with the trajectories of the Li pathways evaluated using the MD simulations. The Li diffusion pathways were optimized until the force on each atom was smaller than 0.05 eV/Å. The bottom two layers of the c-Si slabs were held fixed for all calculations.

## Results and Discussion

### General features of the lithiation behavior of c-Si

#### Lithiation sequence and associated structural evolution

Lithiation of the Si anode begins with the reaction at the interface between the unreacted c-Si and reacted region^15^. Since the diffusion behavior of the Si anode is determined by the characteristics of the interfacial reaction, a simulation technique that can resolve the interfacial reaction occurring at a few atomic layers of c-Si is necessary. Of all the simulation techniques presently available, first-principles calculations provide the most reliable interatomic interactions. However, this method suffers from a severe spatiotemporal limitation, which can cause problems in predicting the Li diffusion pathway and associated energy barrier. For example, first-principles calculations deal with the limited numbers of atoms, usually less than ~200, which makes it difficult to predict the diffusion pathway that are determined from the long-range interatomic interaction. Another problem is that it usually does not consider the temperature effect. These characteristics of first-principles calculations often lead to the prediction of erroneous atomic and electronic structures of a material characterized by long-range atomic interactions and thermal vibrations of the constituent atoms^35^. In order to resolve the initial stage of diffusion at the Li/Si interface while avoiding the problems that arise from first-principles calculations as discussed above, we used a combination of classical MD simulations employing the ReaxFF potential and first-principles calculations; first, the Li diffusion pathway was evaluated in the comparatively large diffusion couples (total ~8400 atoms) at 300 K using MD simulations. The predicted trajectories were then brought into the lattice structures of c-Si generated using first-principles calculations. The energy landscapes corresponding to the Li diffusion trajectories were evaluated via NEB calculations as implemented in Quantum Espresso^33^.

First, we prepared the two different 3D full-scale diffusion couples consisting of a-Li and c-Si using MD simulations, as shown in Fig. 1a and outlined in detail in Methods. In order to explore the orientation dependency of Li diffusion to c-Si, the c-Si slabs were aligned along the <110> and<111> directions so that their (110) and (111) surfaces are in contact with the a-Li layer, constructing the a-Li/Si<110> and a-Li/Si<110> diffusion couples (Fig. 1b and e, respectively). Careful examination of the electrochemical reactions at the interface of the diffusion couples reveal that Li diffusion to c-Si proceeds in two stages (Fig. 1b–g). In the first stage of Li diffusion, Li and Si atoms spontaneously diffuse into each other owing to the chemical potential gradients. It should be noted that first-stage Li diffusion always proceeds through the interstitial sites of a few atomic layers of c-Si without disrupting its initial crystal structure (i.e., diamond cubic), causing the formation of a thin layer of lithiated crystalline Si (hereinafter, this layer is referred to as c-Li_*x*_Si and denoted by the red boxes in Fig. 1c and f). This thin layer of c-Li_*x*_Si is thought to correspond to the amorphous/crystalline interface that was observed experimentally by Liu *at al*.^15^. The c-Li_*x*_Si layer plays an important role in determining the characteristics of the diffusion kinetics. However, the previous models employed for first-principles calculations^22,23^ assumed the reaction in the Li/Si diffusion couple as being between a-Li_*x*_Si and c-Si^23^, and therefore it overlooked the first-stage diffusion at the diffusion front.

Subsequently, Li ions continue to diffuse across the c-Si slab by leaving the lithiated layer behind. During this stage, a continuous supply of Li ions breaks the Si–Si bond of the pre-existing c-Li_*x*_Si, causing the c-Li_*x*_Si layer to evolve into disordered/amorphous lithiated Si (a-Li_*x*_Si) whose volume expands with time (Fig. 1d and g). It is also important to note from Fig. 1 that the transport rate of Li ions to the c-Si side (i.e., the *x*-direction) differs depending on the orientation of c-Si, which is the characteristics of the interface-controlled reaction (see Supplementary Fig. S1). When comparing the results obtained from the two different diffusion couples in Fig. 1, the penetrating rate of the c-Li_*x*_Si layer (equivalently, the rate of Li diffusion) is measurably higher along <110> than along <111> of c-Si. This suggests that the rate of Li diffusion to c-Si is largely determined by first-stage diffusion occurring at the thin layer of c-Li_*x*_Si in the diffusion couples. In order to elucidate the physics underlying the anisotropic lithiation behavior of c-Si, a comprehensive understanding of the first-stage diffusion behavior of Li ions at the c-Li_*x*_Si layer is of great importance.

It is also noted that the metastable a-Li slab begins to crystallize from the free surface side owing to its small dimension/thickness, as revealed by the appearance of the stripes (note that the reflective boundary condition was applied along the *x*-direction to maintain the constant numbers of Li ions in the calculation cell). However, the crystallization of the a-Li slab and its effect on the diffusion behavior are not the subjects of the present study and thus, will not be dealt further in the following.

#### Orientation dependency of Li diffusion to c-Si

**Figure 2:**
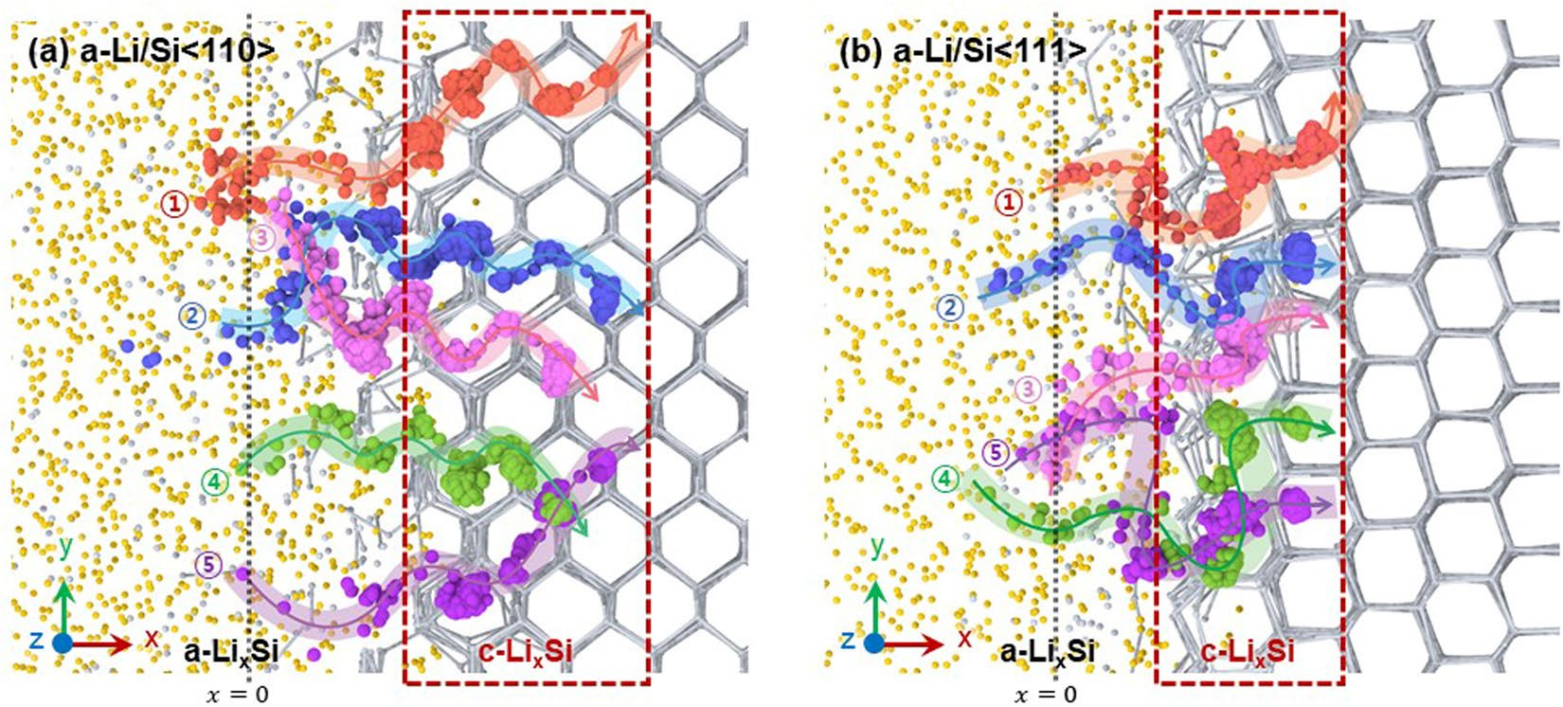
Magnified views of the interfacial region of the (**a**) a-Li/Si<110> and (**b**) a-Li/Si<111> diffusion couples, showing the trajectories of the Li diffusion pathway tracked over 100 ps. Note that the curves highlighted by different colors in (**a**) and (**b**) correspond to the traces of the Li ions that diffused into Si<110> and Si<111> over 100 ps, whereas each sphere comprising the curves corresponds to the positions of the Li ions plotted every 100 fs.

The characteristics of Li diffusion to c-Si were evaluated in more detail by tracking the traces of several arbitrarily chosen Li ions, shown in Fig. 1. Figure 2 displays the diffusion pathways of the Li ions that migrate through the lattice of Si<110> and Si<111>. Li ions, when viewed on a macroscopic scale, diffuse along a direction parallel to the concentration gradient ($\overrightarrow{\nabla }c$) of Li, i.e., the *x*-axis in Fig. 2. However, the atomic-scale view shows that the diffusion pathway of the Li ions is not parallel to $\overrightarrow{\nabla }c$. Rather, they tend to travel in a tortuous manner by jumping from one c-Si interstitial site to another. Furthermore, when comparing the trajectories of the diffusion pathways of the Li ions in Si<110> and Si<111>, the Li ions that diffuse into Si<110> are observed to favor comparatively less winding pathways and thus to travel longer distances. This explains the previous experimental observation that the Li transport rate is faster along <110> than along <111>^4^. The fact that Li ions travel along pathways with different degrees of tortuosity in Si<110> and Si<111> suggests the presence of different energy landscapes along these directions, which may explain why the Li diffusion to c-Si is anisotropic. This concept is thus studied in detail in the following by evaluating the energy landscape corresponding to the diffusion pathways of Li ions that migrate through the c-Li_*x*_Si layer with different orientations.

**Figure 3:**
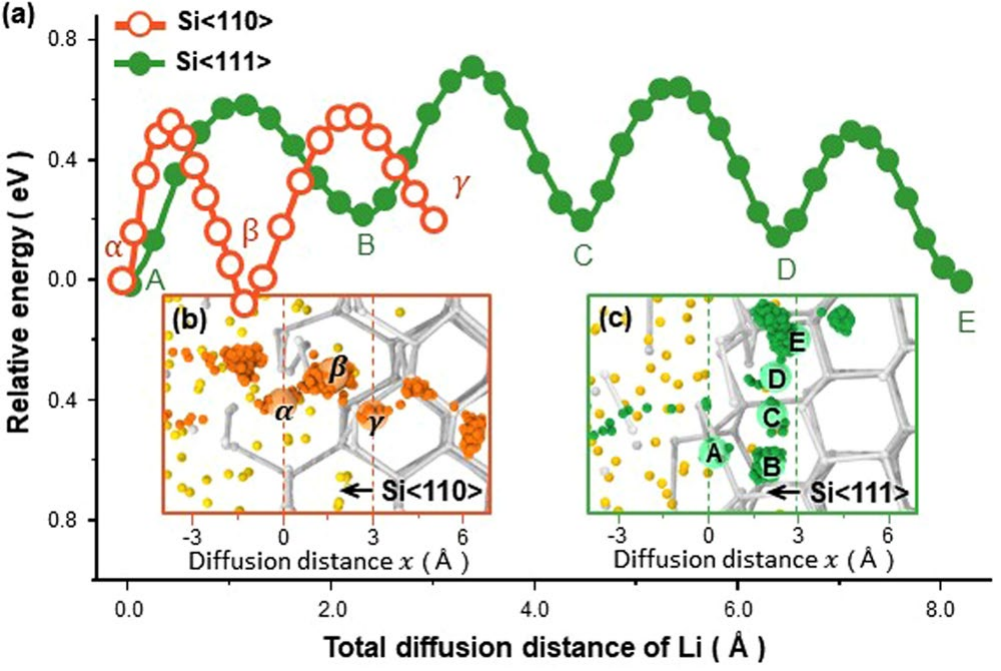
(**a**) Free-energy landscapes corresponding to the diffusion pathways of the Li ions in Si<110> and Si<111> calculated as a function of the total diffusion distance corresponding to the 3D Li diffusion pathway. Trajectories of the Li ions near the interface of the (**b**) a-Li/Si<110> and (**c**) a-Li/Si<111> diffusion couples tracked over 50 ps of lithiation, showing the two different traces of Li pathways used to calculate the free-energy landscapes. Each orange and green sphere corresponds to the positions of the Li ions tracked every 100 fs during diffusion in Si<110> and Si<111>, respectively. Careful examination of (**b**) and (**c**) shows that, in order for Li to migrate a given distance (~3 Å) along the *x*-axis (i.e., the direction parallel to $\overrightarrow{\nabla }c$), the Li atom has to travel a longer total distance in Si<111> than in Si<110>.

In order to evaluate the energy landscape of the Li diffusion pathway and relate its effect on the Li transport rate, we selected the trajectory of a Li atom from each diffusion couple calculated using MD simulations, as shown in Fig. 2 (i.e., atom ② in Fig. 2a and atom ④ in Fig. 2b). We then calculated the changes in the free energy of each atom using the NEB method by moving them along their respective pathways (for details, see First-principles calculations in Methods). Figure 3 displays the energy landscapes along which the Li ions in Si<110> and Si<111> traveled across the c-Li_*x*_Si layer. The values of the energy barriers calculated for Si<110> and Si<111> (Fig. 3a) range from 0.43–0.60 eV. These values are considerably smaller than those evaluated using first-principles calculations alone (0.57–0.79 eV)^36,37^. These differences in the energy barrier values arise from the differences in the calculation methods performed in the present and previous studies; the Li diffusion pathways used for the present calculations were obtained from MD simulations that describe the atomic reactions of a comparatively large-scale sample at 300 K. On the other hand, those used in previous simulation studies were obtained from first-principles calculations performed on a limited sample size at 0 K. This difference in the calculation methods resulted in different Li diffusion pathways; the Li atoms observed in the present study migrates from one interstitial site to another in c-Si without breaking the Si–Si bonds, while those in the previous studies proceeded by breaking the Si–Si bonds^22,23^. The diffusion along the pathway employed in present study is therefore more likely because it is a less-energy-consuming process. Another important finding obtained from the energy landscape analysis in Fig. 3 is that the number of energy barriers for the Li diffusion differs depending on the macroscopic diffusion direction ($\overrightarrow{\nabla }c$), i.e., the orientation of c-Si. As compared to Li ions that diffuse in Si<111>, those that diffuse in Si<110> tend to migrate by taking a less winding path, so that for a given diffusion distance *x*, Li ions will have to overcome a larger number of energy barriers. This energetic interpretation explains the different tortuosity of Li pathways displayed by c-Si with different orientations and thus, clarifies why the Li transport rate in Si<110> is faster than that in Si<111>, confirming the experimental observations reported by Lee *et al*.^3^.

### Quantification of the anisotropic lithiation of c-Si

#### Regions with zero density of valence electrons

Having identified that the anisotropic lithiation behavior of c-Si originates from the different degrees of tortuosity of the Li diffusion pathway (or equivalently, different numbers of energy barriers in the Li diffusion pathway), the next question that naturally arises is why the degree of tortuosity in the Li diffusion pathway differs depending on the orientation of c-Si (i.e., the direction of $\overrightarrow{\nabla }c$). It is well known that the diffusion of Li proceeds according to a concentration gradient by maintaining its macroscopic direction parallel to $\overrightarrow{\nabla }c$. However, when viewed on an atomic scale, Li ions are unable to travel by taking a straight pathway parallel to the direction of $\overrightarrow{\nabla }c$; instead, they have to detour around the lattice of c-Si to avoid colliding with Si atoms. From an electronic perspective, this pathway can be assumed to be a space with zero density of valence electrons (or zero valence electrons, ZVEs) because it allows Li ions to migrate through the surrounding Si atoms by keeping the most stable positions. Under this postulate, the regions corresponding to ZVEs around an atom of a given crystal are determined solely by the species of the atom and its crystal structure. When combining all individual ZVEs of the constituent atoms comprising the crystal, it forms a 3D networked level surface of ZVEs.

**Figure 4:**
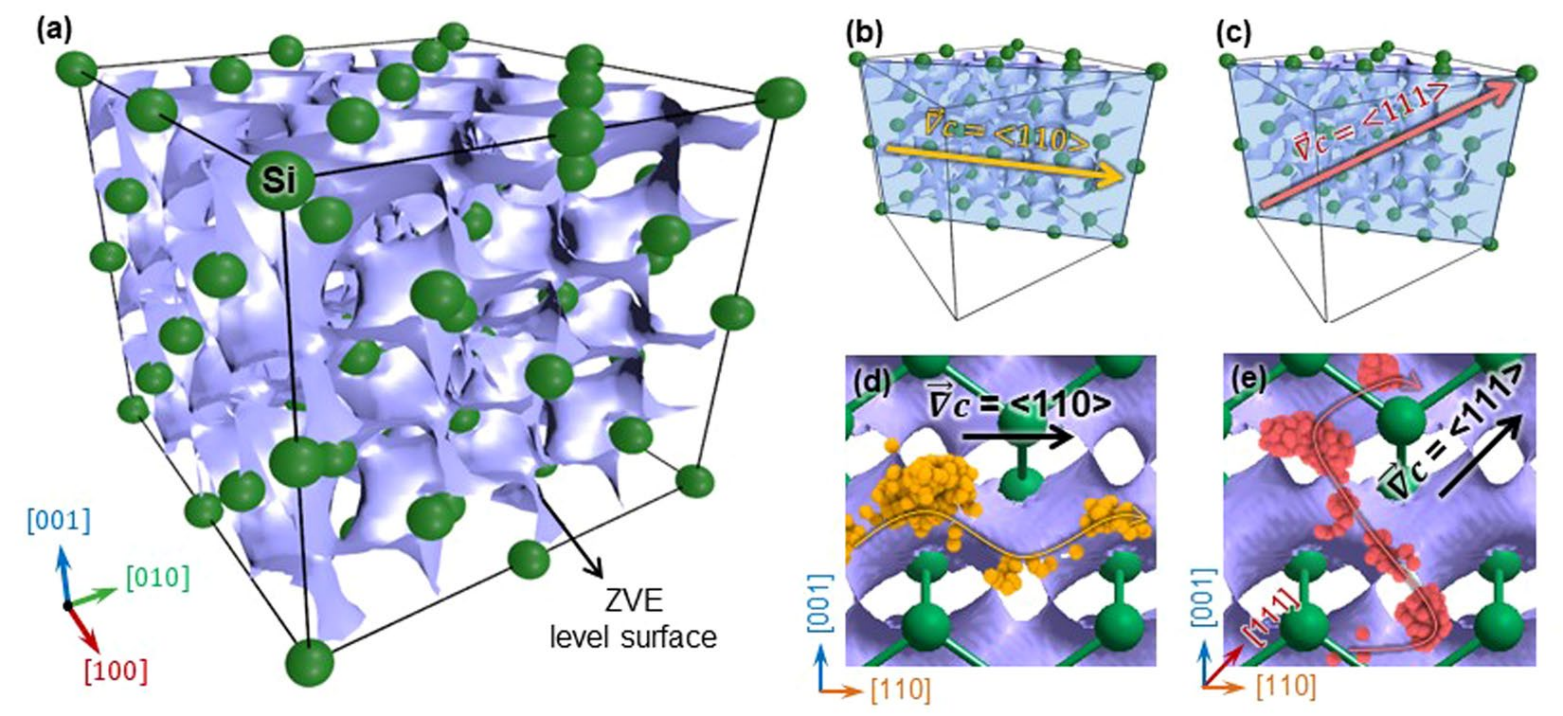
(**a**) ZVE level surface (in blue) superimposed on the supercell structure of c-Si (in green). (**b**,**c**) 2D ZVE surface of c-Si, on which the concentration gradients ($\overrightarrow{\nabla }c$) parallel to (**b**) <110> and (**c**) <111> are denoted. (**d**,**e**) Magnified view of the ZVE surface of (**b**) and (**c**), on which the Li diffusion pathways selected from pathway ② in Fig. 2a and ④ in Fig. 2b are superimposed. Each orange and red sphere corresponds to the positions of the Li ions tracked every 100 fs during diffusion in Si<110> and Si<111>, respectively.

Figure 4a shows the 3D configuration of the ZVE surface superimposed on the supercell structure of c-Si (see Methods for the detailed calculation procedures). Because the 3D configuration of the ZVE surface is determined uniquely by the lattice structure of c-Si, this configuration looks different when viewed from the different crystallographic orientations of c-Si (or the direction of $\overrightarrow{\nabla }c$) (Fig. 4b,c), and thus it can be regarded as the characteristic electronic structure of the material. As such, when Li ions diffuse under the presence of $\overrightarrow{\nabla }c$, the concentration gradient would navigate the Li ions to travel in c-Si by choosing the specific ZVE region whose surface is most parallel to the direction of $\overrightarrow{\nabla }c$. In order to confirm this scenario of diffusion, the trajectories traveled by Li ions under the concentration gradient along <110> and <111> were superimposed on the 2D ZVE surface (Fig. 4d,e). It is clear that the Li ions migrate along the ZVE surface while maintaining their macroscopic diffusion direction parallel to $\overrightarrow{\nabla }c$. However, when comparing the two results, the tortuosity of the diffusion pathways differs depending on the direction of $\overrightarrow{\nabla }c$. This difference in the tortuosity between the Li diffusion pathways is attributed to the different shapes of the ZVE surfaces encountered by the Li ions while traveling along <110> and <111>. This leads us to a conclusion that the ease of Li diffusion into c-Si depends on how parallel the ZVE surface of c-Si is to $\overrightarrow{\nabla }c$.

#### Quantification of anisotropic lithiation

**Figure 5:**
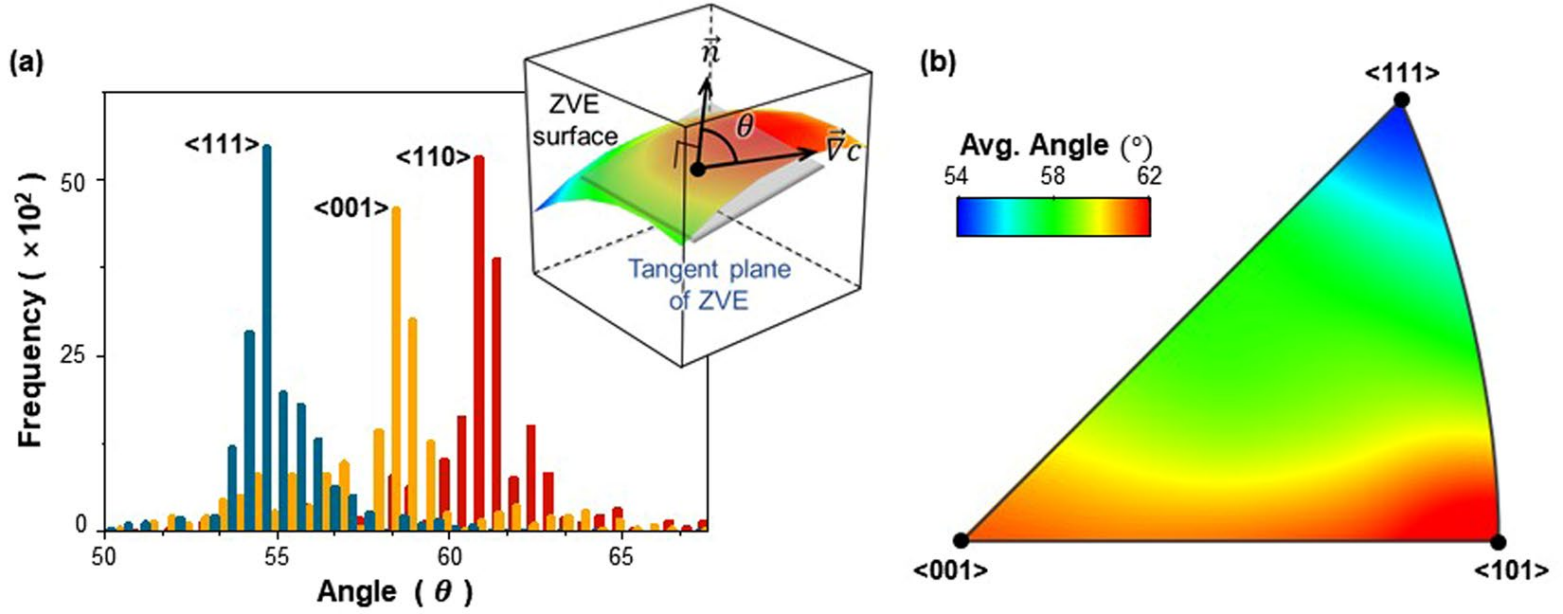
(**a**) Distribution of the angle between the normal vector ($\overrightarrow{n}$) of the ZVE surface and the macroscopic diffusion direction ($\overrightarrow{\nabla }c$) of <111>, <001>, and <110>. The inset in (**a**) is a schematic, showing the relationship between $\overrightarrow{n}$ and $\overrightarrow{\nabla }c$. (**b**) Distribution of the average values of *θ* between $\overrightarrow{n}$ and $\overrightarrow{\nabla }c$ calculated along various crystallographic directions.

Referring back to Fig. 4a, the morphology of the ZVE surface is characterized by the 3D winding surface, meaning that the surface normal vectors are oriented at arbitrary angles with respect to the fixed direction of $\overrightarrow{\nabla }c$. Because the angle between $\overrightarrow{\nabla }c$ and surface normal vector is related to the Li transport rate, i.e., the diffusivity, we evaluated the distribution of the angle of the ZVE surface (*θ*) with respect to a given $\overrightarrow{\nabla }c$. For this purpose, we divided the ZVE surface in Fig. 4a into ~30,000 triangular patches and evaluated the angles of every patches of the ZVE surface for a given direction of $\overrightarrow{\nabla }c$ (see Supporting Information for the detailed calculations). Figure 5a shows the distribution of the *θ* values measured between the ZVE surface of c-Si and three different directions of $\overrightarrow{\nabla }c$ oriented along <110>, <001>, and <111>. Upon comparing the three results, the average *θ* value of the ZVE surface is comparatively greater along $\overrightarrow{\nabla }c$ = <110> than along $\overrightarrow{\nabla }c$ = <001> and <111>. This indicates that the ZVE surface of c-Si is more parallel to <110>, which opens a comparatively less tortuous pathway for Li diffusion. Calculations of the angle between the ZVE surface and $\overrightarrow{\nabla }c$ were then extended to the various crystallographic orientations to obtain the general features for the dependency of the *θ* value of the ZVE surface on the crystallographic orientation (Fig. 5b). Of all the average *θ* values calculated for the various crystallographic orientations, the *θ* value evaluated along <110> was the greatest, followed by those for $\overrightarrow{\nabla }c$ = <001> and <111>. This analysis based on the angle between the ZVE surface and $\overrightarrow{\nabla }c$ is consistent with the results obtained from the previous experimental observations^2–4^ and simulations results^22,23^, which showed that the Li ion diffuses most rapidly along <110>. The fact that the *θ* value is directly related to how well the Li ion diffuses into c-Si thus allows quantification of the diffusivity in terms of the *θ* value.

**Figure 6:**
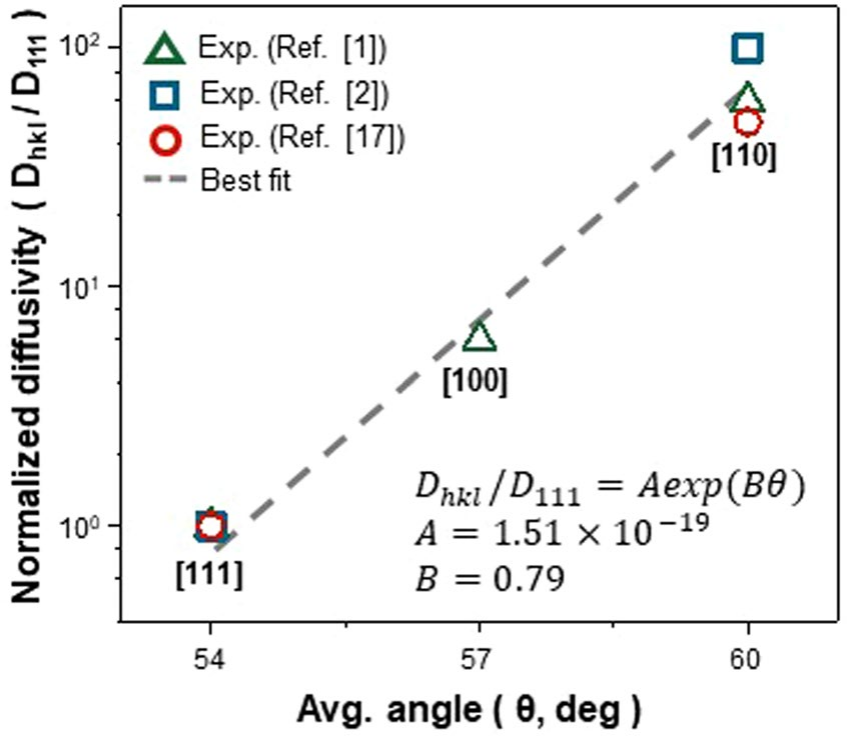
Variations in the Li diffusivity evaluated for various orientations as a function of the average *θ* value. All values of the Li diffusivity are normalized by that measured along <111>. Different symbols denoted in the graph are used to distinguish the values obtained from different sources.

The quantification of the diffusivity in terms of the *θ* value was achieved by comparing the *θ* values evaluated along directions in Fig. 5 with the experimentally measured diffusivities^1,2,22^. The results in Fig. 6 show that the Li diffusivity into c-Si increases exponentially with increasing *θ* value and is given by the canonical equation of

1 $${D}_{ < hkl > }/{D}_{ < 111 > }=Aexp(B{\rm{\theta }})$$

where *A* and *B* are constants with values of 1.51 × 10^−19^ and 0.79, respectively. When comparing the values of the Li diffusivity, it is higher along <110> than along <111> by approximately two orders of magnitude. This implies that the rate of the Li transport is ~10 times faster along <110> than along <111>, which well supports the previous experimental observations^2–4^.

In summary, simulations based on MD and density functional theory (DFT) show that the lithiation of c-Si proceeds by two-stage diffusion. Of the two diffusion processes, the first-stage diffusion is characterized by the penetration of Li ions through the interstitial sites of c-Si by following the ZVE surface of c-Si without disrupting the crystallinity of c-Si. The configuration of the ZVE surface encountered by Li ions during the first-stage diffusion differs depending on the orientation of c-Si, resulting in different Li transport rates. For example, when c-Si is aligned along <110> so that Li ions can diffuse into c-Si along its <110> direction, the rate of the Li transport in LIBs can be enhanced as much as ~10 times. Systematic future work is also required to probe whether the role of the ZVE surface on the diffusivity can be generically applied to other cubic crystals.

## Conclusions

In conclusion, using multi-scale simulations based on classical MD simulations and first-principles calculations, we performed a comprehensive study to elucidate how and why Li diffusion in 3D c-Si is anisotropic. MD simulations resolved the detailed processes of the two-stage Li diffusion in the a-Li/Si<110> and a-Li/Si<111> diffusion couples. During the first stage of lithiation, Li ions tend to migrate through the interstitial sites of c-Si by forming a thin layer of c-Li_*x*_Si without disrupting the initial crystallinity of c-Si. This interfacial reaction occurring at a few atomic layers of c-Li_*x*_Si controls the speed of lithiation. The continuous supply of Li ions forces the ions to penetrate c-Si, during which the Si–Si bond of the pre-formed c-Li_*x*_Si breaks to form a new a-Li_*x*_Si layer.

The interpretation of the MD simulations and the first-principles calculations shows that Li ions tend to travel along a direction parallel to $\overrightarrow{\nabla }c$ by taking a tortuous pathway corresponding to the ZVE surface to benefit from the energy. The configuration of the ZVE surface encountered by the Li ions during first-stage diffusion in c-Si differs depending on the orientation of c-Si, resulting in different Li transport rates under a given $\overrightarrow{\nabla }c$. Based on this finding, we develop a structural parameter (i.e., the *θ* value) that can quantitatively evaluate the degree of the anisotropy of the Li diffusion. By comparing the *θ* value to the diffusion rates evaluated using experiments performed along various orientations of c-Si, we reveal that the configuration of the valence orbital of c-Si is a dominant factor determining the anisotropic lithiation.

## Electronic supplementary material

Supplementary Information

## References

[1] Yang H (2012). Orientation-dependent interfacial mobility governs the anisotropic swelling in lithiated silicon nanowires. Nano Lett..

[2] Liu XH (2011). Anisotropic swelling and fracture of silicon nanowires during lithiation. Nano Lett..

[3] Lee SW, McDowell MT, Choi JW, Cui Y (2011). Anomalous shape changes of silicon nanopillars by electrochemical lithiation. Nano Lett..

[4] Goldman JL, Long BR, Gewirth AA (2011). & Nuzzo, R. G. Strain Anisotropies and Self-Limiting Capacities in Single-Crystalline 3D Silicon Microstructures: Models for High Energy Density Lithium-Ion Battery Anodes. Adv. Funct. Mater..

[5] Sternad, M., Forster, M. & Wilkening, M. The microstructure matters: breaking down the barriers with single crystalline silicon as negative electrode in Li-ion batteries. *Sci. rep*. **6** (2016). 10.1038/srep31712 PMC4987657 27531589

[6] Lee SW, McDowell MT, Berla LA, Nix WD, Cui Y (2012). Fracture of crystalline silicon nanopillars during electrochemical lithium insertion. P. Natl. Acad. Sci..

[7] Magasinski A (2010). High-performance lithium-ion anodes using a hierarchical bottom-up approach. Nat. Mater..

[8] Cui L-F, Ruffo R, Chan CK, Peng H, Cui Y (2008). Crystalline-amorphous core− shell silicon nanowires for high capacity and high current battery electrodes. Nano Lett..

[9] Ikonen, T. *et al*. Electrochemically anodized porous silicon: Towards simple and affordable anode material for Li-ion batteries. *Sci. Rep*. **7** (2017). 10.1038/s41598-017-08285-3 PMC5554169 28801555

[10] Chen C-Y (2016). *In situ* scanning electron microscopy of silicon anode reactions in lithium-ion batteries during charge/discharge processes. Sci. rep..

[11] Mehrer, H. *Diffusion in solids: fundamentals, methods, materials, diffusion-controlled processes*. Vol. 155 (Springer Science & Business Media, 2007).

[12] Cui Z, Gao F, Qu J (2013). Interface-reaction controlled diffusion in binary solids with applications to lithiation of silicon in lithium-ion batteries. J. Mech. Phys. Solids.

[13] Yang H (2014). A chemo-mechanical model of lithiation in silicon. J. Mech. Phys. Solids.

[14] McDowell MT, Lee SW, Wang C, Nix WD, Cui Y (2012). Studying the kinetics of crystalline silicon nanoparticle lithiation with *in situ* transmission electron microscopy. Adv. Mater. (Weinheim, Ger.).

[15] Liu XH (2012). *In situ* atomic-scale imaging of electrochemical lithiation in silicon. Nat. Nanotechnol..

[16] Zhao K (2012). Concurrent reaction and plasticity during initial lithiation of crystalline silicon in lithium-ion batteries. J. Electrochem. Soc..

[17] Tachikawa H, Nagoya Y, Fukuzumi T (2010). Density functional theory (DFT) study on the effects of Li+ doping on electronic states of graphene. J. Power Sources.

[18] Tachikawa H (2007). Diffusion Dynamics of the Li Ion on C60: A Direct Molecular Orbital− Molecular Dynamics Study. J. Phys. Chem. C.

[19] Tachikawa H, Shimizu A (2005). Diffusion dynamics of the Li+ Ion on a model surface of amorphous carbon: a direct molecular orbital dynamics study. J. Phys. Chem. B.

[20] Malyi O, Kulish VV, Tan TL, Manzhos S (2013). A computational study of the insertion of Li, Na, and Mg atoms into Si (111) nanosheets. Nano Energy.

[21] Jin, M., Yu, L., Shi, W., Deng, J. & Zhang, Y. Enhanced Absorption and Diffusion Properties of Lithium on B, N, VC-decoratedGraphene. *Sci. Rep*. 6 (2016). 10.1038/srep37911 PMC5126578 27897202

[22] Cubuk ED (2013). Morphological evolution of Si nanowires upon lithiation: A first-principles multiscale model. Nano Lett..

[23] Jung SC, Choi JW, Han Y-K (2012). Anisotropic volume expansion of crystalline silicon during electrochemical lithium insertion: an atomic level rationale. Nano Lett..

[24] Jung H, Yeo BC, Lee K-R, Han SS (2016). Atomistics of the lithiation of oxidized silicon (SiO x) nanowires in reactive molecular dynamics simulations. Phys. Chem. Chem. Phys..

[25] Jung H, Lee M, Yeo BC, Lee K-R, Han SS (2015). Atomistic observation of the lithiation and delithiation behaviors of silicon nanowires using reactive molecular dynamics simulations. J. Phys. Chem. C.

[26] Reed A, Van Rague Schleyer P, Janoschek R (1991). Why does SiLi4 assume a nontetrahedral (C2v) structure with apparent Li-Li attraction? Comparison with GeLi4, SnLi4, and CLi4. J. Am. Chem. Soc..

[27] Kim H, Chou C-Y, Ekerdt JG, Hwang GS (2011). Structure and properties of Li− Si alloys: a first-principles study. J. Phys. Chem. C.

[28] Bedrov D, Smith GD, Van Duin AC (2012). Reactions of singly-reduced ethylene carbonate in lithium battery electrolytes: a molecular dynamics simulation study using the ReaxFF. J. Phys. Chem. A.

[29] Kim KJ, Wortman J, Kim S-Y, Qi Y (2017). Atomistic Simulation Derived Insight on the Irreversible Structural Changes of Si Electrode during Fast and Slow Delithiation. Nano Lett..

[30] Plimpton S (1995). Fast parallel algorithms for short-range molecular dynamics. J. Comput. Phys..

[31] Perdew J, Burke K, Ernzerhof M (1996). Phys Rev Lett 77: 3865. Errata:(1997) Phys. Rev. Lett..

[32] Kresse G, Joubert D (1999). From ultrasoft pseudopotentials to the projector augmented-wave method. Phys. Rev. B.

[33] Troullier N, Martins JL (1991). Efficient pseudopotentials for plane-wave calculations. Phys. Rev. B.

[34] Henkelman G, Uberuaga BP, Jónsson H (2000). A climbing image nudged elastic band method for finding saddle points and minimum energy paths. J. Chem. Phys..

[35] Friesner RA (2005). Ab initio quantum chemistry: methodology and applications. Proc. Natl. Acad. Sci. USA.

[36] Canham, L. Properties of Silicon. *Electronic Materials Information Service (EMIS)*, 455 (1988).

[37] Milman V (1993). Free energy and entropy of diffusion by ab initio molecular dynamics: Alkali ions in silicon. Phys. Rev. Lett..

