# Supplementary material for: Interfacial Reactions in the Li/Si diffusion couples: Origin of Anisotropic Lithiation of Crystalline Si in Li–Si batteries
Source: Sci Rep. 2017 Oct 25;7:14028. doi: 10.1038/s41598-017-14374-0 (PMC5656666; doi:10.1038/s41598-017-14374-0)
Supplement: Supplementary file 1 — Supplementary Information [file 41598_2017_14374_MOESM1_ESM.pdf]

Supporting Information

Interfacial Reactions in the Li/Si diffusion couples: Origin of Anisotropic Lithiation of Crystalline Si in Li–Si batteries

Yong-Seok Choi[1,§], Jun-Hyoung Park[1,§], Jae-Pyoung Ahn[2], and Jae-Chul Lee[1,*]

[1] Department of Materials Science and Engineering, Korea University, Seoul 136-701, South Korea

[2] Advanced Analysis Center, Korea Institute of Science and Technology, Seoul 02792, South Korea

*To whom correspond should be addressed, E-mail: jclee001@korea.ac.kr

[§] These authors contributed equally to this work.

1. Identification of the diffusion kinetics of the Li-Si diffusion couple

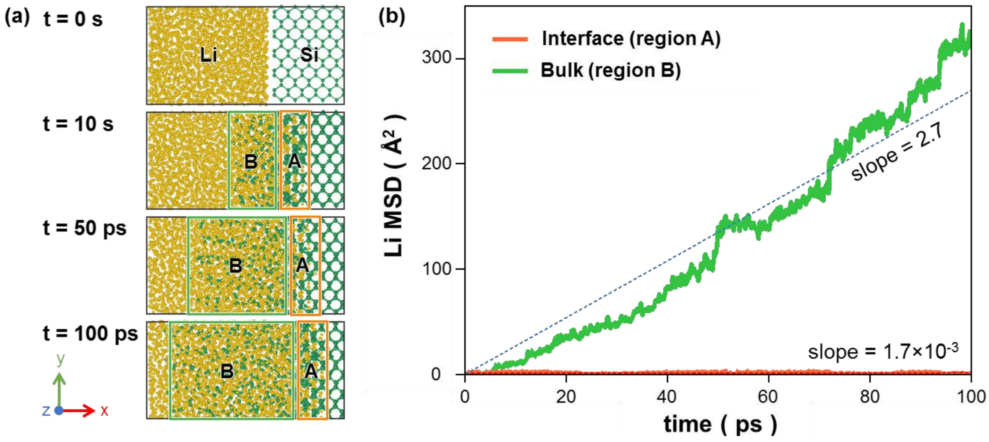

Fig. S1. (a) Magnified views near the interface of the Li/Si diffusion couple captured from molecular dynamics (MD) simulations employing the reactive force field (ReaxFF) potential. The diffusion of Li occurs in two stages; Li^+ first migrates through interstitial sites in the Si lattice without disrupting the initial crystallinity of Si and thereby forms a thin layer of c-Li_xSi (see region A in Fig. S1a). Continuous supply of Li^+ breaks the Si-Si bonds of the pre-existing c-Li_xSi, causing the c-Li_xSi layer to evolve into a-Li_xSi (see region B in Fig. S1a). (b) Mean square displacement (MSD) of Li calculated at regions A and B of (a). The calculated MSD values show that, as lithiation continues, the MSD values at region A becomes much lower than that at region B, suggesting that the diffusion of Li is governed by the interface-controlled reaction. When the calculated MSD values are converted to the diffusivity using the Einstein relation, the diffusivity of Li^+ at region A (2.83×10^{-8} cm^2 s^{-1}) is lower than that at region B (4.50×10^{-5} cm^2 s^{-1}) by more than three orders of magnitude. In these figures, the interfacial and bulk regions formed during lithiation are denoted by the orange and green boxes, respectively. For detailed calculation procedures, see Methods.

Method: Calculation of MSD and diffusivity of Li in the Li/Si diffusion couple.

The positions of the ith Li atom ($R_i(t)$) at time t were tracked during lithiation to evaluate the MSD. The corresponding mean square displacements ($<r^2>$) of the Li atoms were calculated. To obtain the statistically meaningful result under a small number of atoms, the $<r^2>$ values were calculated using the time-averaged mean square displacement [S1,2]. This value was obtained by averaging the mean square displacement over the all choices of particles and time origins, which is given by

$$<r^2(t)> = \frac{1}{N \times n_t} \sum_{t_0=0}^{n_t} \sum_{i=1}^{N} \left(R_i(t_0 + t) - R_i(t_0)\right)^2 \qquad \text{(S1)}$$

where t_0 is the time origins, n_t the total number of t_0, N the total number of atoms. $R_i(t_0)$ and $R_i(t_0 + t)$ are the positions of the ith Li atom at the time origin t_0 and then after time t, respectively. The D_{Li} values at elevated temperatures were evaluated using the Einstein relation:

$$D_{\mathrm{Li}} = \frac{1}{6}\frac{d}{dt}<r^2(t)> \qquad \text{(S2)}$$

Although the diffusivity assessed from Eq. S2 may not be a well-defined quantity from the statistical mechanics perspective, it is useful in determining the relative values of the diffusivity evaluated along various crystallographic orientations [S3,4].

2. Calculation of the θ value between the ZVE surface and $\vec{\nabla}c$

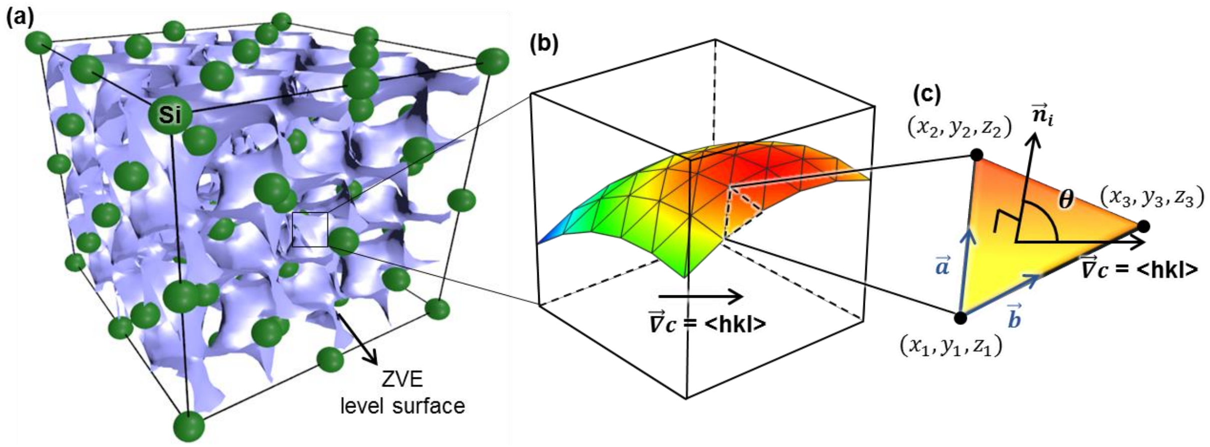

Fig. S2. (a) ZVE level surface (in blue) superimposed on the supercell structure of c-Si (in green). (b) Magnified image of the square denoted in (a), in which the region is again divided into small triangular patches. (c) Schematic of the ith patch defined by the three coordinates of (x_1, y_1, z_1), (x_2, y_2, z_2), and (x_3, y_3, z_3), on which the relationship between the normal vector of the patch ($\vec{n}_i$) and the direction of $\vec{\nabla}c$ is denoted.

Because the morphology of the ZVE surface is 3D winding, the normal vectors of the 3D ZVE surface are oriented arbitrarily for a fixed direction of $\vec{\nabla}c$ (Fig. S2a). In order to evaluate the distribution of the angle (θ) of the ZVE surface with respect to the given $\vec{\nabla}c$, we divided the ZVE surface into >30,000 triangular patches (Fig. S2b) and calculated the θ value for each patch. The θ value (θ_i) of an arbitrary patch can be calculated by evaluating the angle between the normal vector of the patch ($\vec{n}_i$) and the given $\vec{\nabla}c$ (Fig. S2c). The normal vector ($\vec{n}_i$) of the patch can be determined from the three spatial coordinates corresponding to the apex of the triangular patch, i.e., (x_1, y_1, z_1),

(x_2, y_2, z_2), and (x_3, y_3, z_3). Given these coordinates, the two vectors ($\vec{a}$ and $\vec{b}$) parallel to the patch can be obtained by

$$\vec{a} = [x_2 - x_1, y_2 - y_1, z_2 - z_1] \tag{S3}$$

$$\vec{b} = [x_3 - x_1, y_3 - y_1, z_3 - z_1] \tag{S4}$$

The vector $\vec{n}_i$ normal to the triangular patch is obtained by

$$\vec{n}_i = \vec{a} \times \vec{b} = \begin{pmatrix} i & j & k \\ x_2 - x_1 & y_2 - y_1 & z_2 - z_1 \\ x_3 - x_1 & y_3 - y_1 & z_3 - z_1 \end{pmatrix}. \tag{S5}$$

The angle θ_i between $\vec{n}_i$ and $\vec{\nabla} c$ is then determined from the inner product of $\vec{n}_i$ and $\vec{\nabla} c$.

$$\theta_i = \cos^{-1} \frac{\vec{n}_i \cdot \vec{\nabla} c}{|\vec{n}_i||\vec{\nabla} c|} \tag{S6}$$

The average angle θ between the ZVE surface of c-Si and the diffusion direction of $\vec{\nabla} c$ (or equivalently, the crystallographic orientation of Si) is obtained by

$$\theta = \frac{1}{N} \sum_{i=1}^{N} \theta_i, \tag{S7}$$

where N is the number of patches comprising the ZVE surface. Eq. (S7) states that the ZVE surface tends to align parallel to $\vec{\nabla} c$ as the average θ value approaches 90°. Therefore, the average θ value quantifies how parallel the ZVE surface is aligned along the direction of the chemical potential gradient ($\vec{\nabla} c$) responsible for the macroscopic direction of the Li diffusion. As such, for a given crystallographic orientation of Si, the Li transport rate can be assessed by simply evaluating the average θ value according to Eq. (S7).

REFERENCES

[S1] L. Barnard & D. Morgan. Ab initio molecular dynamics simulation of interstitial diffusion in Ni–Cr alloys and implications for radiation induced segregation. *J. Nucl. Mater.* **449** (2014) 225-233.

[S2] B. B. Karki, B. Bohara & L. Stixrude. First-principles study of diffusion and viscosity of anorthite (CaAl2Si2O8) liquid at high pressure. *Am. Mineral.* **96** (2011) 744-751.

[S3] Z. Wang, Q. Su, H. Deng, W. He, J. Lin & Y. Q. Fu. Modelling and simulation of electron-rich effect on Li diffusion in group IVA elements (Si, Ge and Sn) for Li ion batteries. *J. Mater. Chem. A* **2** (2014) 13976-13982.

[S4] P. Johari, Y. Qi & V. B. Shenoy. The mixing mechanism during lithiation of Si negative electrode in Li-ion batteries: an ab initio molecular dynamics study. *Nano Lett.* **11** (2011) 5494-5500.
